# A Viral Noncoding RNA Complements a Weakened Viral RNA Silencing Suppressor and Promotes Efficient Systemic Host Infection

**DOI:** 10.3390/v8100272

**Published:** 2016-10-04

**Authors:** Alyssa Flobinus, Kamal Hleibieh, Elodie Klein, Claudio Ratti, Salah Bouzoubaa, David Gilmer

**Affiliations:** 1 Institut de Biologie Moléculaire des Plantes, Integrative Virology, CNRS UPR2367, Université de Strasbourg, 12 rue du Général Zimmer, 67084 Strasbourg, France; alyssa.flobinus@ibmp-cnrs.unistra.fr (A.F.); kamalhleibieh@gmail.com (K.H.); elodie.klein@ibmp-cnrs.unistra.fr (E.K.); salah.bouzoubaa@ibmp-cnrs.unistra.fr (S.B.); 2 SESVanderHave, Industriepark soldatenplein, Z2nr15, Tienen B3300, Belgium; 3 Dipartimento di Scienze Agrarie, Area Patologia Vegetale, Università di Bologna, Viale Fanin 40, 40127 Bologna, Italy; claudio.ratti@unibo.it

**Keywords:** BNYVV, RSS, RNA silencing suppression, systemic movement, viral noncoding RNA

## Abstract

Systemic movement of beet necrotic yellow vein virus (BNYVV) in *Beta macrocarpa* depends on viral RNA3, whereas in *Nicotiana benthamiana* this RNA is dispensable. RNA3 contains a coremin motif of 20 nucleotides essential for the stabilization of noncoding RNA3 (ncRNA3) and for long-distance movement in *Beta* species. Coremin mutants that are unable to accumulate ncRNA3 also do not achieve systemic movement in *Beta* species. A mutant virus carrying a mutation in the p14 viral suppressor of RNA silencing (VSR), unable to move long distances, can be complemented with the ncRNA3 in the lesion phenotype, viral RNA accumulation, and systemic spread. Analyses of the BNYVV VSR mechanism of action led to the identification of the RNA-dependent RNA polymerase 6 (RDR6) pathway as a target of the virus VSR and the assignment of a VSR function to the ncRNA3.

## 1. Introduction

In eukaryotic cells, antiviral host defenses counteract virus amplification and cell-to-cell transmission. The RNA silencing machinery acts against viral amplification and virus systemic movement in plants [1] and is triggered by the occurrence of double-stranded RNA (dsRNA), which can consist of viral replicative intermediates or highly structured RNA. Dicer-like proteins DCL4 or DCL2 process double-stranded RNAs into 21- to 22-nt-long primary small interfering RNAs (siRNAs), respectively. The siRNAs associate with Argonaute protein-containing RNA-induced silencing complexes (RISC) and guide the complexes to complementary target RNA for endonucleolytic cleavage or translational repression. In plants, fungi, and worms, the process of RNA silencing is amplified by RNA-dependent RNA polymerases (RDR). These enzymes synthesize dsRNAs from targeted RNAs and further processing of these dsRNAs by DCL proteins leads to the production of secondary siRNAs [2]. A remarkable feature of RNA silencing in plants is its non-cell autonomous aspect. In fact, siRNAs move from cell-to-cell through plasmodesmata and their amplification by RDR allows them to reach the phloem and to move systemically [3]. The ability of plants to spread viral siRNAs systemically may play an essential role in antiviral defense.

Viruses overcome host antiviral RNA silencing through the expression of viral RNA silencing suppressors (VSRs) or the production of noncoding RNAs (ncRNAs) acting as RNA silencing decoys or sponges for cellular factors as described for cauliflower mosaic virus (CaMV) 8S RNA [4,5] or noncoding subgenomic RNAs produced by different viral genus [6]. These VSRs target one or more steps in the antiviral pathway. Tomato bushy stunt virus (TBSV) p19 protein binds 21-nt-long siRNA duplexes and prevents RISC formation [1], whereas Turnip yellows virus (TuYV) P0 protein targets Argonaute 1 (AGO1) to induce its degradation by autophagy [7]. VSR activity of p14 protein of beet necrotic yellow vein virus (BNYVV) has been associated with reduced accumulation of primary and secondary siRNAs [8]. BNYVV belongs to the *Benyviridae* family and is the type member of the *Benyvirus* genus [9]. It is transmitted by the soil-borne protozoa *Polymyxa betae* and causes sugar beet rhizomania disease. The BNYVV genome is composed of four to five linear, positive-sense, single-stranded RNAs that are capped and polyadenylated. RNA1 and RNA2 are essential and sufficient for viral multiplication in rub-inoculated laboratory host plants. RNA1 encodes proteins required for replication, while RNA2 encodes proteins for packaging and cell-to-cell movement, as well as the p14 protein required for RNA silencing suppression and systemic spread of the virus [8]. The smaller RNAs (RNA3, RNA4, and, when present, RNA5) are dispensable for infection of laboratory hosts upon mechanical inoculation but are required for the natural infection of *Beta* species and for transmission of the virus. RNA3 influences symptom expression in host plants through its p25 protein [10,11]. RNA3 is also essential for BNYVV long-distance movement in *Beta* species, but this does not require p25, as p25-deficient RNA3 still moves long distances [12]. The establishment of systemic infection in *Beta macrocarpa* depends on a 20-nt-long coremin sequence [13] embedded in the RNA3 ‘core’ region [12]. Coremin is also found in RNA5 of BNYVV as well as in the small genomic RNAs of the *Benyviridae* species beet soil borne mosaic virus and in two unrelated viral genera [13,14]. Previously, we have shown that RNA3 encodes a subgenomic non-coding RNA (ncRNA3). BNYVV ncRNA3 is produced by nuclease activity and not by the viral polymerase [13]. Mutagenesis of the coremin motif prevents ncRNA3 production and viral systemic spread [13].

The role of ncRNA3 accumulation along the BNYVV life cycle is not fully understood. Since viral systemic spread requires both p14 and ncRNA3, we addressed the synergic effect of both elements by testing the complementation of p14 mutants with wild-type (WT) or mutated RNA3. We show that, in *Nicotiana benthamiana (N. benthamiana)*, the p14BA2 mutant of the VSR unable to move long distances [8] is indeed complemented by RNA3, and that ncRNA3 accumulation plays an essential role in systemic infection, acting as a second VSR. Moreover, our data tend to support the activity of p14 in the inhibition of intercellular siRNA movement, which is mandatory in *Beta* species for BNYVV systemic movement and is enhanced by the production of ncRNA3.

## 2. Materials and Methods

### 2.1. Plasmids

Plasmids encoding RNA1, RNA2 (WT or mutant for p14), RNA3, and RNA3E full-length infectious complementary DNA clones were described previously [8,13,15].

### 2.2. In Vitro Transcription, Plant Infection, Protein, and RNA Extractions

The linearized full-length BNYVV clones of RNA1 (pB15), RNA2 (pB2-14), RNA2Δp14 (pB2-3722), RNA2BA2 (pB2-BA2) [8], RNA3 (pB35), RNA3E (pB35E) served for in vitro run-off transcription as described previously [13,15]. RNA3∆p25 (pB35∆aug) and RNA3E∆p25 (pB35E∆aug) do not allow the expression of the p25 protein as the initiation codon has been removed, as described in [12].

RNA1 and RNA2 (RNA2, RNA2Δp14, or RNA2BA2) species, supplemented or not with RNA3 or RNA3E, served for the mechanical infection of *Chenopodium quinoa* (*C. quinoa*) or WT and knockdown RDR6i *N. benthamiana* leaves [16], or the electroporation of *C. quinoa* protoplasts, as described previously [17,18,19]. RDR6i seeds were kindly provided by D. Baulcombe (Cambridge, UK).

RNAs and proteins were extracted from infected tissues and *C. quinoa* protoplasts. Protein detection was performed by Western blotting using antisera directed against BNYVV coat protein (CP), p14, and p25, as described previously [8,18]. RNAs were extracted using a “polysomes” buffer [20] followed by phenol/chloroform purification and ethanol precipitation. Pellets were treated with a 3 M sodium acetate solution (pH 5.5) (Promega, Madison, WI, USA) to solubilize DNA and small RNAs. Remaining RNA pellets were washed with 70% ethanol before dissolution in RNase-free water. RNAs were extracted from protoplasts after cell lysis in suspension buffer (50 mM Tris-HCl pH 7.5, 1 mM ethylenediaminetetraacetic acid (EDTA; Euromedex, Strasbourg, France), 0.05% macaloïde, 1% sodium dodecyl sulphate (SDS; Euromedex), 150 mM NaCl) followed by two extractions with phenol/chloroform and ethanol precipitation. Northern blots were performed as described previously [13,18].

### 2.3. Agroinfiltration of N. benthamiana 16C

Cultures of *Agrobacterium tumefaciens* cells (strain GV3101) were prepared as described previously [8]. An optical density of 1.5 was used for agroinfiltration in patch test assays [21]. Leaves of the green fluorescent protein (GFP)-expressing *N. benthamiana* line 16C (4–5-week-old seedlings) were co-agroinfiltrated with bacteria transformed with binary vector (based on pBin61) expressing either BNYVV-p14 or mutant p14BA2, TuYV P0, or TBSV p19, or empty vector, together with agrobacteria containing a binary vector encoding GFP messenger RNA (mRNA) to trigger RNA silencing. RNAs and proteins were extracted from agro-infiltrated leaves using TRIzol reagent (Sigma-Aldrich, Saint-Louis, MO, USA) following the manufacturer’s recommendations and a Laemmli buffer [21].

## 3. Results

### 3.1. The ncRNA3 Complements the Absence of BNYVV VSR in *C. quinoa*

**Figure 1:**
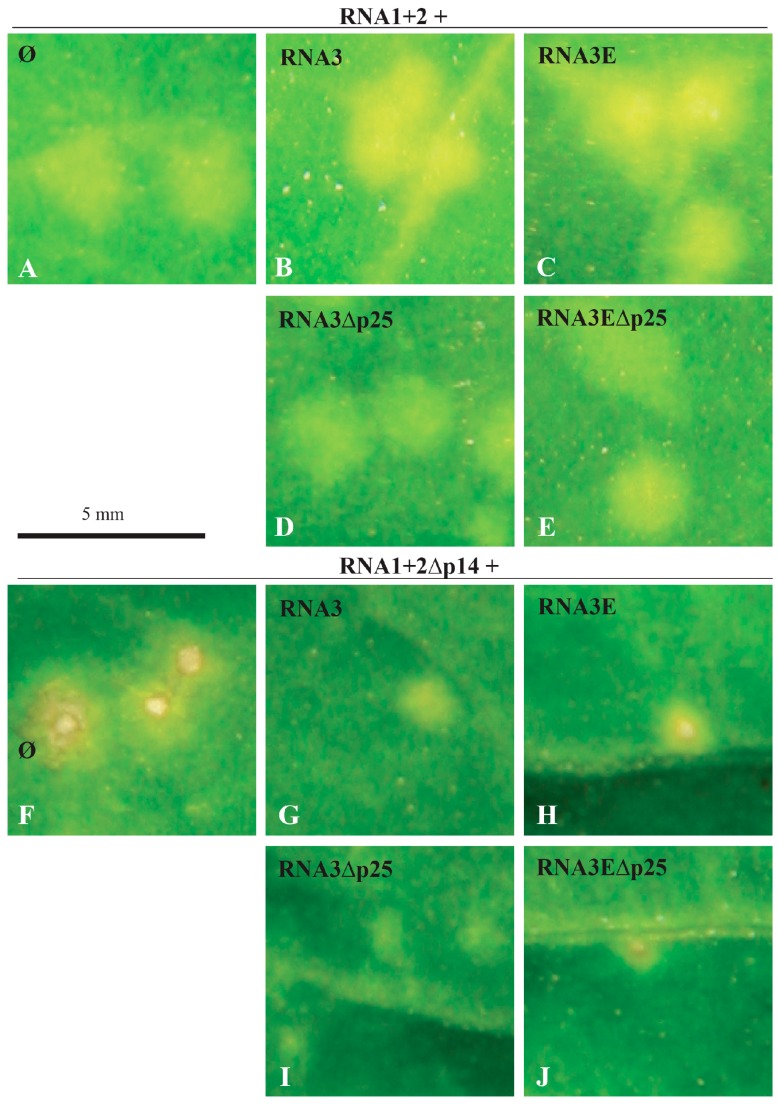
Beet necrotic yellow vein virus (BNYVV) noncoding RNA3 (ncRNA3) complements the absence of the p14 viral suppressor of RNA silencing (VSR) protein independently of expression of the RNA3-encoded p25 protein. The presence of ncRNA3-producing RNA species alleviates the necrosis obtained in the absence of p14. A close-up of *Chenopodium quinoa* local lesions at seven days post-infection (dpi) with genomic RNA1 + 2 (**A**–**E**) or RNA1 + 2∆p14 (deletion of the VSR) (**F**–**J**), in the presence of RNA3 species able to accumulate ncRNA3 (panels **B**, **D**, **G**, and **I**) or in the presence of RNA3E species unable to accumulate ncRNA3 (panels **C**, **E**, **H**, and **J**).

The VSR protein of BNYVV (p14) was shown to be essential for long-distance movement of the virus in *Beta* species and for the systemic movement of the genomic RNA1 and RNA2 in *N. benthamiana* [8]. Upon inoculation of *C. quinoa* leaves, RNA1 + RNA2 are sufficient to produce green chlorotic local lesions (Figure 1A). RNA3 that contains the coremin sequence is able to drive the accumulation of ncRNA3, while RNA3E possesses the reverse complement sequence and does not accumulate ncRNA3 [13]. Coexpression of RNA 1 and RNA 2 with RNA3 or RNA3E causes the spots in the leaves to turn yellow (Figure 1B,C). The lesions remained green when p25-deficient RNA3 or RNA3E were present (Figure 1D,E; RNA3∆p25 and RNA3E∆p25, respectively). Inoculation of leaves with RNA1 and p14-deficient RNA2 (p14null mutant, 1 + 2∆p14) leads to the production of small necrotic lesions (Figure 1F). Interestingly, when RNA3 was present, small chlorotic spots were observed without noticeable changes in lesion size (Figure 1G,I). Conversely, no phenotypic change was observed when RNA3E and RNA3E∆p25 were inoculated (Figure 1H,J). This indicates that the symptom attenuation was due to the production of the ncRNA3.

We have previously shown that RNA2 encoding the p14BA2 mutant (KK^78–79^AA) induces small chlorotic local lesions possessing a necrotic center [8]. Moreover, this mutant is not able to support systemic movement of the virus in *N. benthamiana* despite its residual VSR activity [8]. When we applied the same complementation study to the p14BA2 mutant, we observed lesions lacking the necrotic center only in the presence of RNA3 species producing the ncRNA3 (data not shown). This shows that ncRNA3 prevents the induction of necrosis.

**Figure 2:**
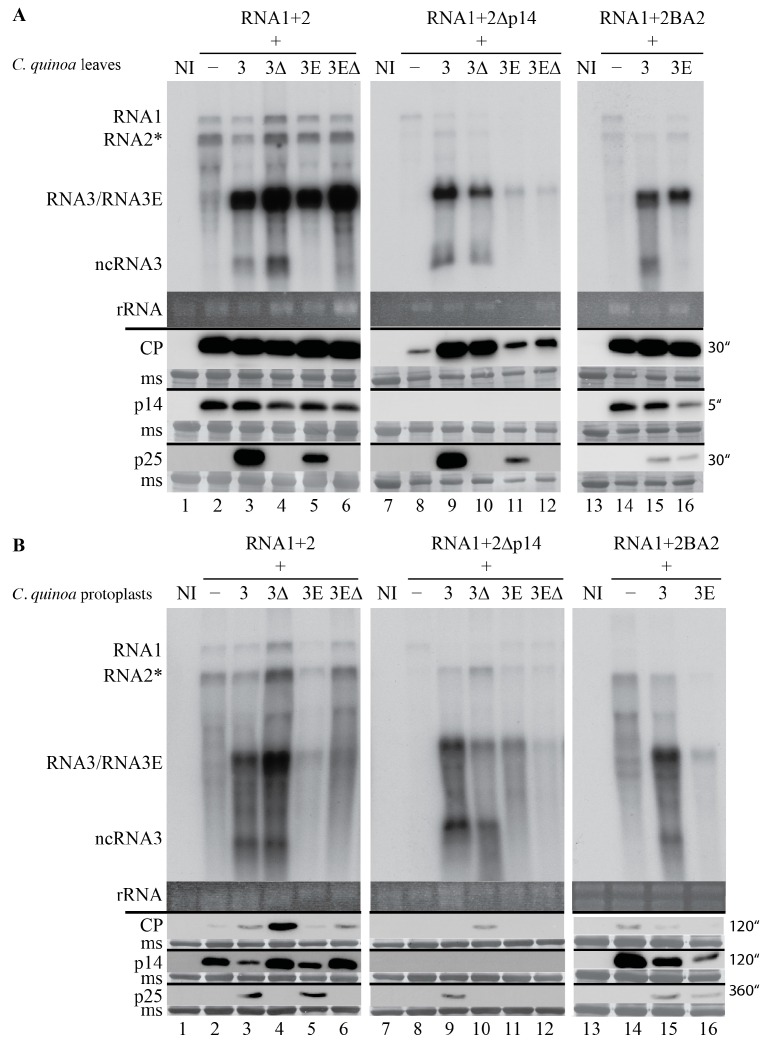
RNAs producing ncRNA3 species partially restore viral RNA and protein accumulation, which is lower in the absence of the p14 VSR or in the presence of the p14BA2 mutant. (**A**) *C. quinoa* local lesion at 7 dpi and (**B**) *C. quinoa* protoplast RNA and protein at 40 h post-infection were extracted and analyzed by Northern blot (upper panels) using BNYVV-specific RNA probes, and Western blot (lower panels) using anti-coat protein (CP), anti-p14 and anti-p25 sera. Exposure times for protein detections are indicated. Inoculum consisted of RNA1 + RNA2 (left panels), RNA1 + RNA2∆p14 (middle panels) or RNA1 + RNA2BA2 (right panels) alone (−) or supplemented by wild-type RNA3 (3), RNA3p25null mutant (3Δ) or the same RNA species unable to produce ncRNA3 (3E and 3EΔ). The position of the RNA species is indicated on the left. Ethidium bromide staining of ribosomal RNA (rRNA) and membrane staining (ms) were used as loading controls. Protoplast infection was performed once. Depending on the RNA combination, leaf infections were conducted twice or more and gave comparable results. NI: non-infected.

We analyzed the seven-day-old local lesions and searched for variation of viral expression either at the RNA or protein levels (Figure 2A). To ensure a correct interpretation of the data obtained, *C. quinoa* protoplast infection experiments were conducted in parallel, and samples were harvested 40 h post-infection (Figure 2B). The viral ncRNA3 species were produced in all combinations containing either RNA3 or mutant RNA3∆p25 (3 or 3∆ respectively), both in leaves and in protoplasts, irrespective of which RNA2 species was used (Figure 2A,B; lanes 3, 4, 9, 10, and 15).

**Figure 3:**
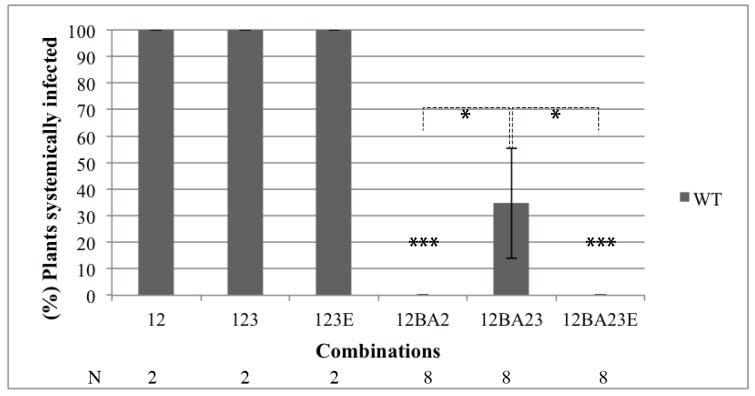
The BNYVV ncRNA3 contributes to systemic movement of the BA2 mutant of the BNYVV VSR. Wild-type (WT) *Nicotiana benthamiana* were infected with RNA1 + 2 (12) or RNA1 + 2BA2 (12BA2) supplemented or not with RNA3 (123, 12BA23) or RNA3E (123E, 12BA23E). Viral RNA detection on upper leaves was performed at 21 dpi. Three independent experiments using specified numbers (N) of plants were performed, and statistical analyses were carried out using Fisher’s exact test. The *p*-values were obtained from the false discovery rate method. * *p*-value < 0.05 and *** *p*-value < 0.001.

In local lesions, RNA1 + RNA2 displayed comparable RNA1 and RNA2 accumulation levels and similar amounts of CP and p14 proteins in the absence of the p25 protein (Figure 2A; 3∆ and 3E∆; compare lanes 2, 4, and 6). The expression of the p25 protein entailed a faint reduction of genomic RNAs, an increased expression of CP, and no variation in the expression of p14 (Figure 2A; 3 or 3E; lanes 3 and 5). Mutation of the coremin sequence did not affect RNA3E accumulation in leaves (Figure 2A, compare lane 3 to lane 5 or lane 4 to lane 6); however, p25 protein expression was reduced in the absence of ncRNA3 (Figure 2A, lanes 3 and 5, p25). In the absence of the VSR (RNA1 + 2∆p14), the accumulation of genomic RNA1 and RNA2 was severely compromised, and the CP was barely detectable in local lesions (Figure 2A, lane 8). The presence of ncRNA3 favored RNA1 and RNA2 species accumulation and enhanced CP accumulation in local lesions independently of p25 (Figure 2A, lanes 9 and 10). Conversely, the presence of RNA3 mutant species (3E and 3E∆) had a negative effect on genomic RNA and CP accumulations (Figure 2A, compare lanes 11–12 to lanes 9–10). Accumulation of RNA1 + RNA2BA2 (Figure 2A, right panel) ranged between WT and p14-null mutant, and CP expression appeared unchanged (Figure 2A, lanes 14–16). We noticed that the absence of ncRNA3 caused a lower expression of p25 for a yet unknown reason (Figure 2A, compare lanes 3 to 5, 9 to 11, and 15 to 16).

Protoplast infection can be compared to a “one-step growth curve” usually described for bacterial viruses [22]. In this cell-autonomous system, both genomic RNA and p14 protein levels were lower when p25 was expressed (Figure 2B, compare lanes 3 and 5 to lanes 2, 4, and 6). In the absence of p14 (Figure 2B, central panel), replication of RNA3 impaired RNA1 accumulation (Figure 2B, lanes 9–12), and production of ncRNA3 increased RNA2 accumulation, but the CP was only detectable in the absence of p25 (Figure 2B, compare lanes 9 and 10). In the absence of ncRNA3, the CP was below detection limit (Figure 2B, lanes 8, 11, and 12). The behavior of RNA1 + RNA2BA2 mutant in protoplasts was as in leaves, albeit with a lower protein accumulation. The results obtained in protoplasts corroborated those obtained through the analysis of local lesions.

Taken together, these observations on the *C. quinoa* host indicated that RNA3 is able to complement lesion phenotype and to some extent restore viral expression through production of ncRNA.

### 3.2. The ncRNA3 Promotes Systemic Movement of p14BA2 VSR Mutant in *N. benthamiana*

As stated above, BNYVV systemic movement in *Beta* species requires both RNA2-encoded p14 and RNA3, whereas in *N. benthamiana* RNA3 is dispensable but p14 protein expression remains absolutely required [8]. Since ncRNA3 production was able to complement p14-BA2 VSR mutant in infected cells and tissues, we asked whether the presence of the ncRNA3 could rescue RNA1 + RNA2BA2 long-distance movement [8]. Plants were inoculated with combinations of RNA1 supplemented with WT or RNA2BA2 species alone or combined with RNA3 or RNA3E. Systemic infection was monitored at 21 days post-infection (dpi) by Northern blot analyses performed on total RNAs extracted from upper leaves. Plants infected with RNA1 + RNA2 (namely 12) supplemented with RNA3 (123) or RNA3E (123E) were systemically infected (Figure 3), while RNA1 + RNA2BA2 (namely 12BA2) was not able to move in the absence of ncRNA3 production (Figure 3, 12BA2 and 12BA23E). However, when ncRNA3 was produced, it provided significant complementation of the systemic movement in more than 30% of the plants inoculated with the mutant expressing p14BA2 (Figure 3, 12BA23) (*p* < 0.05, false discovery rate method of Fisher’s exact test). When RNA1 + RNA2∆p14 was used, no systemic movement was observed even in the presence of RNA3 (data not shown), confirming the absolute requirement of the p14 protein for the long-distance spread of the virus.

### 3.3. Silencing of *N. benthamiana* RDR6 Allows Systemic Movement of VSR BA2 Mutant Independently of the Presence of RNA3

**Figure 4:**
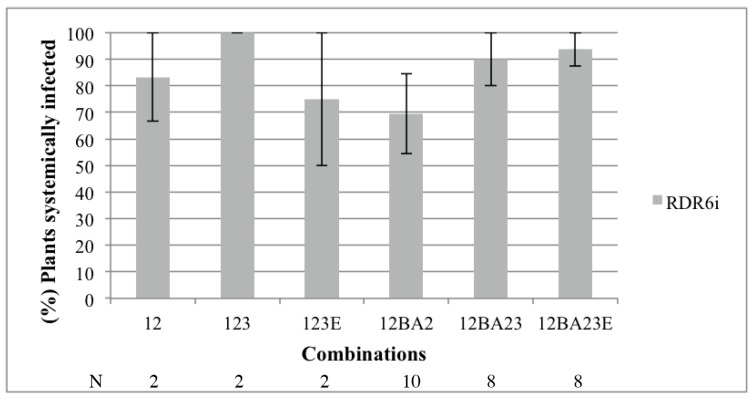
Systemic movement of BNYVV expressing BA2 p14 mutant is restored by a RNA-dependent RNA polymerase 6 knockdown (RDR6i). RDR6i *Nicotiana benthamiana* were infected with RNA1 + 2 (12) or RNA1 + 2BA2 (12BA2) supplemented or not with RNA3 (123, 12BA23) or RNA3E (123E, 12BA23E). Viral RNA detection on upper leaves was performed at 21 dpi. Three independent experiments using specified numbers (N) of plants were performed, and statistical analyses were carried out using Fisher’s exact test.

Since p14 was shown to reduce secondary siRNA production in agroinfiltration patch experiments [8], we wanted to determine whether this protein interferes with RDR6 as presumed previously [23,24]. We thus applied inocula of RNA1 supplemented with RNA2 or RNA2BA2 in the presence of RNA3 or RNA3E to RDR6 knockdown plants (RDR6i) and monitored systemic infection. *N. benthamiana* RDR6i plants were inoculated and analyzed similarly as described above. RNA1 + RNA2∆p14 was not able to move in RDR6i upper leaves, even in the presence of RNA3 (data not shown). Interestingly, all viral RNA combinations were able to move independently of the presence of RNA3 (Figure 4). Statistical analyses using Fisher’s exact test and false discovery rate did not highlight significant differences between the inocula (*p*-value > 0.05), even if the presence of RNA3 slightly increased the systemic infection. We concluded from this experiment that the knockdown of RDR6 complemented p14BA2 long-distance movement deficiency. Taken together, the results obtained using WT and RDR6i plants indicated a functional role of ncRNA3 on the BNYVV VSR as the presence of RNA3 complemented partially the VSR mutant (compare Figure 3 and Figure 4, 12BA2 and 12BA23). This also suggests that p14 inhibits the effect of the RDR6 pathway, presumably by acting on the production or systemic movement of secondary siRNAs as stated above.

### 3.4. BNYVV p14 Inhibits the Systemic Spread of RNA Silencing

**Figure 5:**
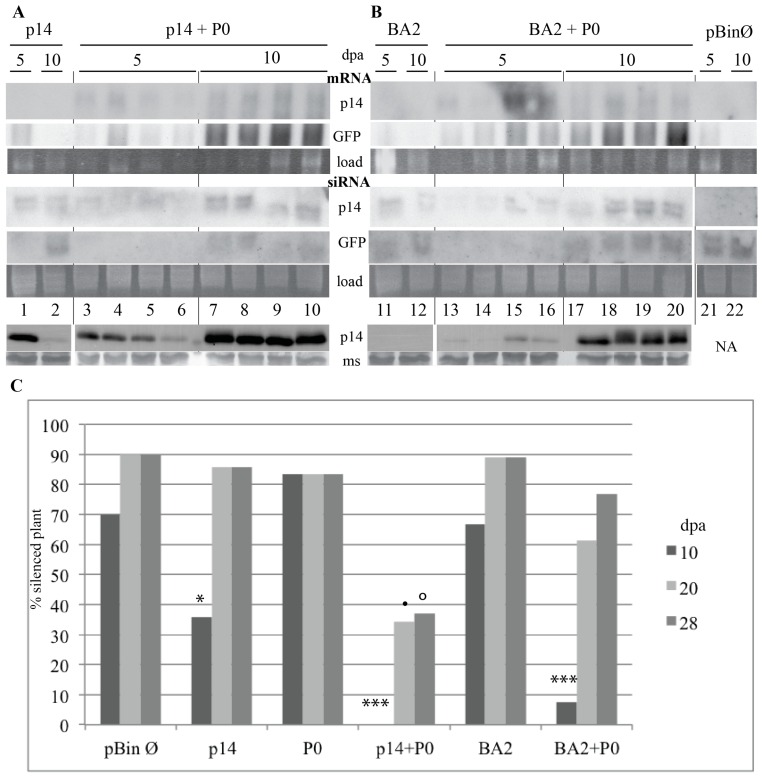
Stabilization of BNYVV VSR ectopic expression reveals its inhibitory effect on systemic movement of RNA silencing in *N. benthamiana* 16C. The expression of p14 in patch tests leads to poor expression, while their co-expression with P0 stabilizes the mRNA and maintains p14 protein function. Plants were infiltrated with a mixture of *Agrobacterium tumefaciens* bacteria carrying a binary vector allowing the expression of the GFP trigger and p14 (**A**) or its mutant BA2 (**B**) alone (lanes 1–2 and 11–12, respectively) or with turnip yellows virus P0 (p14 + P0, lanes 3–10; BA2, lanes 13–20) or in the presence of the empty vector (**B**, pBinØ lanes 21–22). Individual patches were collected at 5 and 10 days post-agroinfiltration (dpa) from infiltrated areas. p14, BA2, and GFP mRNA (upper panels) and siRNA accumulations (middle panels) were tested via Northern blot. p14 and BA2 were detected via Western blot (lower panels). Total proteins were visualized by membrane staining (ms). Spaces between lanes 6–7 and 16–17 correspond to molecular weight markers positions. RNA loading is visualized by ethidium bromide staining (load); (**C**) The histogram represents the percentage of plants systemically silenced for each combination. The systemic movement of RNA silencing was assessed under an ultraviolet (UV) lamp using plants infiltrated with empty vector (Ø, 4 experiments, 10 plants) as a control after 10, 21, and 28 days. Systemic silencing was delayed in the presence of p14 + P0 (4 experiments, 25 plants) and started to appear for some of the plants at 20 dpa and thus differed from p14 (4 experiments, 12 plants) or P0 (3 experiments, 9 plants) alone (p14 + P0, 20 dpa; *p*-value < 0.05). The stabilization of p14BA2 (3 experiments, 13 plants) decreased systemic silencing compared with BA2 (3 experiments, 9 plants) or P0 alone, and few plants started to present systemic silencing after 10 days (BA2 + P0, 10 dpa; *p*-value < 0.001). After 20 days, systemic silencing was as the controls (BA2 + P0, 20/28 dpa; *p*-values > 0.3 compared with P0 or BA2 alone). Statistical analyses were performed using Fisher’s exact test. The *p*-values were obtained using the false discovery rate method. (*, •, and o correspond to 10, 20, and 28 dpa, respectively). *p*-value < 0.05 and *** *p*-value < 0.001. NA: not applicable.

Agroinfiltration patch tests suggested that p14 could inhibit intercellular silencing signaling (this study and [8]) similarly to tombusvirus p19 [25]. The expression of p14 in the patches peaked 4–5 days post-agroinfiltration (dpa) and then decreased rapidly. As shown in Figure 5, the p14 mRNA was not detectable, while p14-specific siRNAs were found. The GFP mRNA was detected at 5 dpa but disappeared at 10 dpa when GFP siRNAs appeared (Figure 5A, p14, lanes 1–2). In the absence of p14, GFP siRNAs were detected at 5 and 10 dpa (Figure 5B, lanes 21–22). GFP mRNA was only detected at 5 dpa (Figure 5B, lane 21). When the p14BA2 mutant was expressed, reduced accumulation of GFP mRNA and higher amounts of GFP siRNAs were detected at 5 dpa, and the p14BA2 protein was below the detection limit (Figure 5B, BA2, lanes 11–12).

p14 is not stable when expressed outside its viral context [8], and its accumulation decreases rapidly (Figure 5A, lanes 1–2). Therefore, we concluded that this transient expression system does not reflect the situation occurring during genuine viral infection. Indeed, during infection, p14 is constitutively expressed. To stabilize p14 mRNA in infiltrated tissues, we decided to co-express p14 in combination with polerovirus P0, a VSR that acts by inducing degradation of Argonaute proteins and does not inhibit the movement of siRNAs [7,26,27]. Using this approach, p14 and p14BA2 protein expression lingered for 10 days (Figure 5A,B, bottom). This was due to the stabilization of the mRNAs (Figure 5A, lanes 3–10 and Figure 5B, lanes 13–20). At 5 dpa, low amounts of anti-GFP and anti-p14 siRNAs were found in the patches but started to accumulate at 10 dpa (Figure 5A, p14 + P0, lanes 3–10); the same observations were made with the p14BA2 mutant (Figure 5B, BA2 + P0, lanes 13–20). Therefore, co-expression of these VSRs did not interfere with the effect of p14, p14BA2, or P0 on the reduction of siRNAs (Figure 5) or secondary siRNA production (data not shown). We concluded that the expression of P0 was able to grant continuous production of p14 under transient expression conditions.

We evaluated the movement of the silencing signal on 16C plants infiltrated with different combinations of *A. tumefaciens* strains containing binary vectors expressing the GFP trigger, an empty vector, and a vector encoding the VSR (p19, P0, p14, or p14BA2). We monitored GFP fluorescence of the patch and of upper leaves for 28 days (data not shown) as described [27]. Ten days after agroinfiltration, we observed a red fluorescent area around the agroinfiltrated patch, indicating the spread of RNA silencing from the agroinfiltrated cells into non-agroinfiltrated cells when the empty binary vector, or a vector expressing P0 or p14BA2, was used. Systemic silencing was delayed when p14 was present (Figure 5C, p14, 10 dpa; *p*-value < 0.05) and was suppressed in the presence of p19 (data not shown) as described [25]. Thus, unlike p19, neither p14 nor P0 suppressed systemic silencing signaling. Interestingly, the red area around infiltration patches seen at 5 dpa was absent when both p14 and P0 proteins were expressed together (data not shown) and subsequent systemic movement of silencing was delayed (Figure 5C, p14 + P0, 10 dpa; *p*-value < 0.001 and 20/28 dpa; *p*-value < 0.05). Taken together, these results indicate that p14 is able to interfere with the production or with the movement of the silencing signal when its expression is stabilized.

## 4. Discussion

The BNYVV RNA3 “core” and particularly the 20-nt coremin motif are RNA sequences required for ncRNA3 accumulation and the systemic spread of the virus in *B. macrocarpa* species [12,13,14]. The coremin motif resides in the RNA3 core sequence and is involved in stalling exoribonucleases (5’-3’ exoribonuclease 1 (Xrn1) and exoribonuclease 4 (XRN4) in yeast and Xrn1 in vitro) that lead to the production of ncRNA3 [28]. The BNYVV p14 VSR protein expression is also essential for the systemic spread of the virus in *Beta* species and in *N. benthamiana* [8], although in this latter host, the presence of RNA3 is not required for long-distance movement. In the absence of a functional VSR, no complementation of the systemic spread by ncRNA3 is observed. This indicates that either p14 and ncRNA3 act on separate pathways of the RNAi machinery or the strength of ncRNA3 suppression of RNA silencing alone is not sufficient. In such a situation, BNYVV fails to counteract the plant defense silencing machinery and the viral infection remains limited to small necrotic lesions. BNYVV encoding the BA2 mutant of p14 produces small local lesions with a necrotic center in *C. quinoa* and fails to move systemically in *N. benthamiana* [8]. Partial complementation of this local lesion phenotype was observed when RNA3 species able to produce ncRNA3 were used (as shown in Figure 1 for p14-null mutant), since ncRNA3 increased the levels of genomic RNAs and viral proteins particularly for the BA2 mutant (Figure 2). Consequently, the presence of p14 or ncRNA3 increases viral amplification in local tissues. The synergistic effect of ncRNA3 on silencing suppression mediated by p14 was confirmed through complementation of the BA2 mutant by ncRNA3-producing species (Figure 3). We conclude that ncRNA3 acts as a second VSR. Production of ncRNA3 alleviated viral restriction of the BA2 mutant (that fails to inhibit secondary siRNA production [8]) within primary infected cells, probably through direct or indirect suppression of RNA silencing. As secondary siRNAs are the product of RDR6-mediated transitivity [1,2], we tested the systemic spread of RNA1 + RNA2BA2 mutant in *N*. *benthamiana* RDR6-silenced plants. In a RDR6i host, the RNA1 + RNA2BA2 mutant was able to move to the distal part of the plant even in the absence of ncRNA3 (Figure 4). From these experiments, we conclude that p14 affects the RDR6 pathway, as does tomato chlorosis virus p22 VSR [29], and that ncRNA3 plays a role in this mechanism.

BNYVV infection of GFP-silenced *N. benthamiana* 16C restores the expression of the GFP protein in the infected tissues [8]. However, patch test experiments involving p14 and p14BA2 ectopic expression, because of impaired accumulation of these proteins, did not reveal efficient inhibition of siRNA systemic movement by p14 (Figure 5). This ectopic expression profile is far from the accumulation levels of p14 during infection. We stabilized p14 expression in the presence of TuYV-encoded P0 VSR, which does not interfere with siRNA systemic spread [26,27]. This approach allowed us to clearly demonstrate that p14 is able to block the systemic movement of siRNAs (Figure 5).

Taken together, our previous analyses [8], and these results demonstrate the high efficiency of BNYVV p14 in blocking secondary siRNAs production via the RDR6 pathway. However, the role of ncRNA3 has not been thoroughly assessed. This ncRNA is produced in the absence of RNA3 replication [13] as does red clover necrotic mosaic virus small RNA derived from RNA1 (SR1f RNA) [30] by the action of 5′ to 3′ exoribonuclease [28] as described for subgenomic flavivirus RNAs (sfRNAs) [31]. Interestingly, ncRNA3, as sfRNAs, does not interfere with viral RNA amplification but increases viral pathogenicity and inhibits RNA interference (RNAi) [31,32]. Moreover, other viral long ncRNA involved in the inhibition of the silencing machinery have been described for adenoviruses virus-associated RNAs (VA-RNAs) [33] and plant viruses [4,5,6]. These ncRNA species modulate cellular or viral translation or accumulation efficiencies by highjacking cellular proteins to prevent their function, as reviewed in [6].

## 5. Conclusions

BNYVV long-distance movement requires effective expression of its p14 VSR, which is in turn enhanced by the accumulation of ncRNA during the infection. BNYVV p14 acts on RDR6-dependent transitivity, while ncRNA3 accumulation acts as a second VSR that directly or indirectly targets the silencing machinery. Considering the link between the exoribonuclease (Xrn) requirement for the production of ncRNA3 and its synergistic effect on the p14BA2 mutant, a clear evolutionary role could be assigned to the viral ncRNAs produced to modulate antiviral host responses, a situation not unique to BNYVV, as stated above. Further experiments will aim to identify the cellular factors bound to ncRNA3 and p14. However, as *A. thaliana* is not a host for BNYVV and no genetic tool is yet available for *Beta* species or *N. benthamiana* to set up screens, conventional biochemistry approaches will be needed to further investigate these viral effectors, which act both on the suppression of RNA silencing and on viral systemic movement.
